# Dealing with coronavirus disease 2019 (COVID-19) outbreaks in long-term care homes: A protocol for room moving and cohorting

**DOI:** 10.1017/ice.2020.1302

**Published:** 2020-10-28

**Authors:** Dylan C. Kain, Liz J. McCreight, Jennie Johnstone

**Affiliations:** 1 Department of Medicine, University of Toronto, Toronto, Canada; 2 Infection Prevention and Control, Sinai Health, Toronto, Canada; 3 Department of Laboratory Medicine and Pathobiology, University of Toronto, Toronto, Canada; 4 Dalla Lana School of Public Health, University of Toronto, Toronto, Canada

*To the Editor—*As coronavirus disease 2019 (COVID-19) has swiftly moved across the world, it has had an especially large impact in long-term care homes, with many countries reporting >50% of COVID-19 related deaths due to outbreaks in long-term care home.^1^ Outbreaks in these homes can rapidly spread with high mortality,^2^ and homes with multiple residents per room have been forced to move residents within the home to slow and prevent further spread. Such movements may contribute to the larger impact of COVID-19 in homes with increased multiple-bed rooms.^3^ In most long-term care homes, moving people from their room within the home for the purpose of infection control was a completely novel concept. Consequently, those responsible for these room movements often did not have experience with this type of movement. To our knowledge, no existing guidance on principles of room movement is available for long-term care homes for the purposes of infection control.^4^

When our infection control team was partnered with several long-term care homes across the Toronto area, we identified this knowledge gap and worked to create a guidance with the goal of providing a tool to help homes with multiple-bed rooms work through logical moves to reduce the risk of COVID-19 transmission (Table 1). Priority of resident moves and cohorting are a key consideration because homes often have limited space. In an outbreak, cleaning staff may not be able to clean rooms at the pace needed to make all room moves simultaneously. Having a better understanding of principles of room movement during a COVID-19 outbreak setting may help minimize the size and scale of the outbreaks in the first wave.

**Table 1.** Principals of Room Movements in Long-Term Care Homes During the COVID-19 Pandemic

Steps After Identifying a Symptomatic Resident
1. Immediately move the symptomatic resident^5^ to a private room on precautions for COVID-19^a^ and place the remaining residents that have been exposed on precautions for COVID-19. If possible move each exposed resident to their own private room; if not feasible, keep the exposed residents within the room, and maintain precautions at the bedside.
2. At a minimum test the symptomatic resident as well as the exposed roommates for COVID-19.
3. In the event any of the test results are positive for COVID-19, all exposed residents must be on appropriate precautions for 14 days from their last exposure to the positive resident. Ideally residents exposed to a positive resident will be moved to a private room, regardless of their RT-PCR results.
4. A diagnosis of COVID-19 in a resident of a home, should prompt consideration of a COVID-19 outbreak. Further testing and suspected outbreak measures should be implemented at the direction of public health authorities.
Guideline for Cohorting Residents
1. The fewest room moves possible should be made that ensure safety of residents. Movement is labor intensive; it generates possible risk of exposure; and it can distress residents.
2. Asymptomatic exposed residents without confirmed COVID-19 should never be isolated in a cohort together.^b^
3. Residents with confirmed COVID-19 during the same outbreak can be cohorted together if necessary, to allow cleaning and freeing additional space within the home.
4. If an exposure has occurred the asymptomatic exposed residents should be moved out and placed in a private room on appropriate COVID-19 precautions (from each other and others) because they are potentially already infected and incubating.
5. If at all possible, residents should not be moved from a floor with COVID-19 cases to a floor without COVID-19 cases (regardless of the individuals COVID-19 swab status).

a In Canada, COVID-19 precautions include droplet and contact precautions, but local guidance should be followed.

b High-risk exposure defined as sustained (>15 min), unmasked contact with a confirmed or suspected COVID-19 case during the 2 d prior to symptom onset until day 8 after symptom onset.^6^

As we prepare for a second wave of disease in many countries, having clear guidance for homes on ways to safely move residents is critical to preventing such large-scale outbreaks. We hope this guidance will serve as a template for long-term care homes moving forward.
